# Biliary Adenofibroma: A Rare Liver Tumor with Transition to Invasive Carcinoma

**DOI:** 10.1155/2022/5280884

**Published:** 2022-02-07

**Authors:** Ayham Alshbib, Krzysztof Grzyb, Trygve Syversveen, Henrik Mikael Reims, Kristoffer Lassen, Sheraz Yaqub

**Affiliations:** ^1^Department of Hepato-Pancreato-Biliary Surgery, Oslo University Hospital, Oslo, Norway; ^2^Department of Pathology, Oslo University Hospital, Oslo, Norway; ^3^Department of Radiology and Nuclear Medicine, Oslo University Hospital, Oslo, Norway; ^4^Institute of Clinical Medicine, University of Tromsø, Tromsø, Norway; ^5^Institute of Clinical Medicine, University of Oslo, Oslo, Norway

## Abstract

Biliary adenofibroma is a rare benign liver tumor with potential for malignant transition. It has a bile duct origin characterized by a complex tubulocystic biliary epithelium with fibrous stroma. MRI features may suggest this uncommon entity, and histological findings can be diagnostic. We report a case of biliary adenofibroma with transformation to an intrahepatic cholangiocarcinoma.

## 1. Introduction

Biliary adenofibroma is an extremely rare hepatic tumor. It has similar histological components as von Meyenburg complexes and is classified as a benign tumor. However, biliary adenofibromas are larger and have the potential for malignant transformation [1–7]. We report a case of biliary adenofibroma with malignant transformation.

## 2. Case presentation

**Figure 1:**
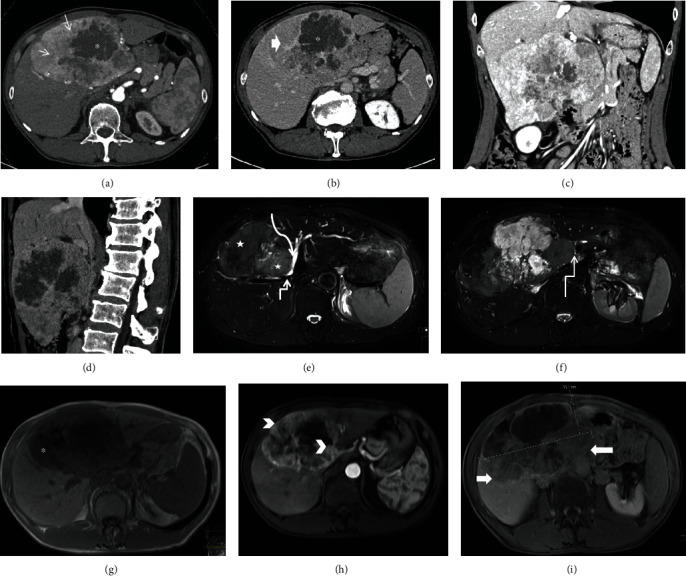
CT scan (a–d) showed a large multiloculated, partly nonvascularized (^∗^), partly hypervascularized (arrows) tumor with washout (thick arrow). MRI (e–i) confirmed a 15.5 × 9.6 × 14.2 cm tumor which was T1 hypointense (^∗^) and heterogeneously T2 hyperintense (stars) and displayed varying levels of peripheral enhancement on postcontrast sequences (arrow heads) with washout (thick arrows). The mass compressed the central and extrahepatic bile ducts (angulated arrows) with dilated intrahepatic bile ducts (curved arrow).

A 63-year-old male patient from the Middle East, who had previous surgery for inguinal hernia, was admitted at a local hospital due to unspecific epigastric pain. Clinical examination revealed a large palpable tumor in the upper part of the abdomen. Blood test showed normal liver parameters. Abdominal computed tomography (CT) scan showed a large multiloculated tumor in liver segments IVb and V with arterial hyperenhancement in the majority of the tumor, partly with washout in the late phase. A nonvascularized central component was interpreted as necrosis (Figure 1). A hepatocellular carcinoma was suspected, and he was referred to our hepatobiliary surgical unit for further evaluation and management. Magnetic resonance imaging (MRI) scans confirmed a 15.5 × 9.6 × 14.2 cm tumor which was T1 inhomogeneous, hypointense, with signal loss in all phases in a part of the tumor consistent with hemorrhagic content, and heterogeneously T2 hyperintense and displayed varying levels of peripheral enhancement on postcontrast sequences with washout (Figure 1). The mass compressed the central and extrahepatic bile ducts with dilated intrahepatic bile ducts. There were no radiological signs of liver cirrhosis, and the tumor was radiographically interpreted as a hepatocellular carcinoma. There was no evidence of hepatic or extrahepatic metastases. The patient had no other concomitant disease. He smoked 20 cigarettes daily. No alcohol consumption was reported. He had no family history of liver disease. The physical examination confirmed a hard palpable mass in the upper mid and right quadrant. The laboratory tests showed no pathological findings except for an increased CA 19-9 concentration of 163 U/mL. Other tumor markers including AFP and CEA were within the reference range. The tumor was excised with anatomical resection of segments IVb and V with margins. His postoperative course was uncomplicated, and he was discharged on postoperative day 7. A new CT scan at follow-up after 3 months revealed a 15 mm lesion in liver segment VI, which on previous scans had measured 8 mm and was interpreted as a benign lesion, but now was highly suspicious for metastasis. FDG-PET CT scan was positive for the lesion in liver segment VI but showed no other metastases. The metastasis was removed with laparoscopic local liver resection, and the patient was discharged on postoperative day 1.

## 3. Pathological findings

### 3.1. Macroscopic findings

The resected specimen containing liver segments IVb and V measured 170 × 130 × 80 mm and weighed 990 grams. At gross examination, the tumor was a solid mass (170 mm), with a pale and spongy cut surface with some areas with small cysts and focal hemorrhage. The subsequent specimen from liver segment VI measured 40 × 35 × 11 mm, weighing 14 grams, and contained a 20 mm firm tumor with a pale cut surface.

### 3.2. Histological Findings

**Figure 2:**
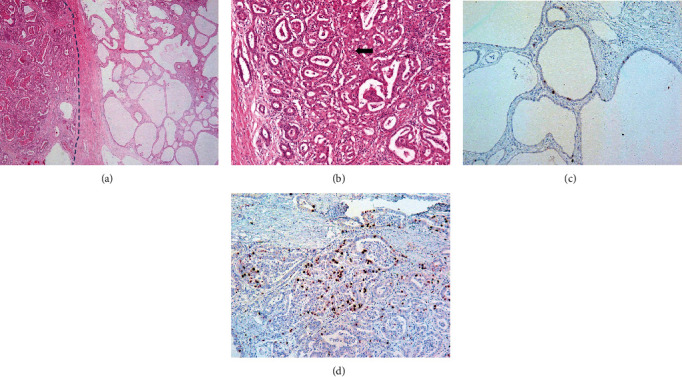
Histological and immunohistochemical examination of the adenofibroma. (a) Hematoxylin and eosin-stained sections of the tumor showing cholangiocarcinoma (left side of line) and adenofibroma with cyst formation (right side) (×400). (b) Adenofibroma with complex architecture showing crowded back-to-back anastomosing and cribriform-like tubules (arrow) (×100). Immunohistochemical staining for Ki67 in adenofibroma (c) and cholangiocarcinoma (d) (×200).

Histologically, the primary tumor was relatively well circumscribed, but in some peripheral areas, irregular glandular structures were found infiltrating into the adjacent parenchyma (Figure 2). The tumor had a combination of tubules and microcysts. The background was collagenous with a variable inflammatory infiltrate. Some areas contained intratumoral hemorrhage with red blood cells in the tubules. Tubules were lined by cuboidal or low columnar epithelium, whereas the epithelium in the cysts was flattened. The epithelial lining cells in the adenofibroma had bland round nuclei with minimal contour irregularity. The carcinomatous component of the tumor showed a complex architecture with crowded back-to-back anastomosing and cribriform-like tubules. In this area, the epithelial lining was cuboidal with prominent nucleoli. The subsequently resected tumor from segment VI was morphologically similar to the carcinoma found within the adenofibroma and thus consistent with metastatic spread.

## 4. Immunohistochemistry and Genetic Analyses

The epithelial lining in both the benign parts of the adenofibroma and in the carcinoma stained positively for CK7, CK20, MUC1, and polyclonal CEA. There was focal positive staining for CD56, but mainly in the carcinoma. Both components had expression of SMAD4 and BAP1. Staining for p53 showed weak nuclear staining in a few individual cells in the adenofibroma and in the carcinoma. No nuclear staining for beta-catenin was observed. Immunohistochemical staining for BRAF V600E mutation (BRAF VE1) was negative.

The Ki67 proliferation index was 9% in the benign areas of adenofibroma, whereas in the carcinoma it was up to 25% (Figure 2).

Molecular genetic analysis in the benign area detected CCND1 gain with a copy number ratio of 10.78 (locus chr11:69455944). No fusion transcripts were detected, including FGFR 1/2/3 or NTRK 1/2/3. In the area with malignant transformation, the CCND1 copy number ratio was 12.54 (locus chr11:69455944), and NRAS mutation (c.181C>A; p.Gln61Lys) was detected. Microsatellite instability (MSI) analysis revealed a microsatellite stabile (MSS) phenotype.

## 5. Discussion

Biliary adenofibroma is a very rare benign liver tumor with, to our knowledge, only 25 cases previously reported in the literature, seven of which showed malignant transformation (Table 1).

The present case showed evidence of invasive carcinoma. This is in line with previously published cases, suggesting a relatively high risk of malignant transformation. However, to our knowledge, only one previously reported case developed metastatic disease at follow-up.

Characteristic MRI-findings of biliary adenofibroma have previously been suggested to be a well-circumscribed multicystic tumor with septal enhancement and no intrahepatic bile duct communication, based on a report of two cases and a review of MRI features reported in the literature [8]. Differential diagnoses for biliary adenofibroma include cystic neoplasms such as intraductal papillary neoplasms of the bile duct (IPNB) and mucinous cystic neoplasm of the liver (MCN) [8].

Our case showed larger solid and less cystic components than in previous reports regarding biliary adenofibroma. An atypical HCC was suspected due to imaging features with a large tumor with HCC enhancement pattern combined with a substantial proportion of cystic/necrotic and additionally hemorrhagic components. With a relatively little proportion being cystic (and additionally interpreted as necrosis), cystic neoplasm was not suspected in our case.

While abnormal nuclear p53 accumulation has been found in some benign adenofibromas, the few studied cases with malignant transformation have been p53 negative or shown focal to moderate positivity (up to 50%) [9]. In the present case, we found only weak and focal nuclear staining for p53 in individual cells. The role of TP53 mutations in biliary adenofibromas and their malignant potential has not been extensively studied, and accordingly, the potential utility of p53 immunohistochemistry has not been defined.

Based on the histological features, the presence of epithelial components with architectural complexity, such as atypical papillary or tubulopapillary growth patterns and back-to-back or cribriform glands, as in our case, may indicate malignant transformation. Similar to the findings in a recently published case series [9], the Ki67 proliferation index was <10 in the epithelial component of adenofibroma in the present case. By contrast, the Ki67 index was 25% in the malignant part of the tumor. Molecular genetic analysis in our case did not reveal any fusion transcripts which would enable the use of FGFR inhibitor or NTRK inhibitor. The role of immunotherapy in the treatment of cholangiocarcinoma is currently under investigation, and checkpoint inhibitors have shown encouraging results in patients with MSI [10]. In the present case, the tumor was MSS, suggesting that treatment with checkpoint inhibitors may not be indicated. We did not stain the tumor for PD-L1, but this may be an option, as checkpoint inhibitors may show efficacy in PD-L1-positive tumors [11].

## 6. Conclusion

We present a rare case of a large biliary adenofibroma with transformation to cholangiocarcinoma. We recommend local resection with free margins and imaging surveillance follow-up for potential recurrence. Molecular tumor profiling may reveal novel treatment options and is recommended for this rare entity.

## Figures and Tables

**Table 1:** Biliary adenofibroma reported in the literature.

No.	Reference	Age/sex	Tumor size (cm)	Ki67 (%)	p53 (%)	Malignant features
1	Tsui et al. [12]	74/F	7.0	NA	NA	No
2	Parada et al. [13]	49/M	7.0	NA	NA	No
3	Akin and Coskun [1]			NA	NA	Metastasis
4	Garduno-Lòpez et al. [14]	68/M	6.0	NA	NA	No
5	Varnholt et al. [15]	21/F	16.0	<10	50-70	No
6	Gurrera et al. [16]	79/M	5.5	1	Negative	No
7	Kai et al. [17]	40/M	7.0	Negative	5-10	Suspicious
8	Nguyen et al. [2]	53/F	6.5	NA	NA	Yes
9	Tsutsui et al. [18]	69/F	3.5	10-15	Focally positive	Suspicious
10	Jacobs et al. [19]	57/F	10.0	NA	NA	Suspicious
11	Thai et al. [3]	77/M	4.8	NA	NA	Yes
12	Godambe et al. [4]	71/F	6.3	50	25-50	Yes
13	Elpek et al. [20]	23/M	6.0	NA	NA	No
14	Thompson et al. [5]	71/M	14.5	NA	NA	Yes
15	Kaminsky et al. [6]	37/F	4.5	50	Negative	Yes
16	Arnason et al. [9]	83/M	7.0	NA	NA	NA
17		47/F	16.0	6	NA	Suspicious
18		57/F	10; 2.5; 1.7	<10	Positive	Suspicious
19		70/F	12.0	<8	Negative	Suspicious
20		74/F	7.0	2	Negative	No
21		46/M	15.0	<1	Patchy positive	Suspicious
22	Esteban et al. [21]	26/F	2.6	NA	NA	No
23	Sturm et al. [7]	63/F	6.5	20-30	Focally positive	Yes
24	Lee et al. [8]	63/M	4.7	<2%	Focally positive	No
25		38/M	2.5	NA	NA	No
26	Alshbib et al. (current case)	63/M	17.0; 2.0	25	Negative	Yes

## Data Availability

The data used to support the findings of this study are included within the article.
